# Transcatheter Paravalvular Leak Closure: A Step-by-Step Guide

**DOI:** 10.3390/jcdd13020096

**Published:** 2026-02-16

**Authors:** Georgios E. Papadopoulos, Ilias Ninios, Sotirios Evangelou, Andreas Ioannides, Vlasis Ninios

**Affiliations:** Cardiology Department, Interbalkan Medical Center, 55535 Thessaloniki, Greece; iliasninios@gmail.com (I.N.); evangelousotirios@yahoo.com (S.E.); ioannidesandreas69@gmail.com (A.I.); v.ninios@interbalkan-hosp.gr (V.N.)

**Keywords:** paravalvular leak, transcatheter closure, prosthetic valve, hemolysis, heart failure, transesophageal echocardiography, cardiac computed tomography, cardiac magnetic resonance, TAVI, complications, bailout strategies

## Abstract

Paravalvular leak (PVL) remains a clinically important complication after surgical or transcatheter valve implantation, presenting predominantly with heart failure (HF) and/or high-shear hemolysis. While redo surgery can be definitive, contemporary candidates frequently carry prohibitive operative risk, positioning transcatheter PVL closure as a key therapeutic alternative. However, available outcome data are largely derived from observational series and registries with heterogeneity in PVL mechanisms, prosthesis types, imaging protocols, and endpoint definitions. Standardized frameworks—such as those proposed by the PVL Academic Research Consortium—support harmonized PVL grading and clinically meaningful composite endpoints that integrate imaging/hemodynamic results with patient-centered outcomes. Across datasets, the most consistent determinant of benefit is residual PVL severity: procedural efficacy is most commonly defined as achieving ≤ mild residual regurgitation without prosthetic leaflet interference, device embolization, or major complications. This review provides a step-by-step, phenotype-driven approach to transcatheter PVL closure, emphasizing multimodality imaging (TEE and cardiac CT, with adjunct CMR and PET when appropriate), access and support planning tailored to valve position, and morphology-matched device selection—often requiring modular multi-device strategies for elongated crescentic channels, particularly in hemolysis-predominant presentations. We also synthesize evidence on complications and bailout management, with a focus on preventable high-severity events (leaflet impingement, embolization, stroke/air, vascular injury, tamponade) and standardized pre-release safety checks. Collectively, contemporary practice supports high implant success in experienced programs, with clinical improvement tightly coupled to procedural endpoint quality and careful Heart Team selection.

## 1. Introduction

Paravalvular leak (PVL) is a prototypical example of how a seemingly “small” structural defect can translate into disproportionately large clinical consequences [1,2]. PVL refers to regurgitant flow between a prosthetic valve and surrounding native tissue, typically caused by incomplete apposition of the sewing ring or valve frame to the annulus or by acquired dehiscence [1,2]. PVL is encountered after surgical valve replacement and, through distinct mechanisms, after transcatheter valve implantation. Its clinical spectrum ranges from incidentally detected trivial jets to severe regurgitation with cardiogenic shock, pulmonary edema, or transfusion-dependent hemolysis [1,2,3]. Importantly, PVL severity by color Doppler alone may underestimate clinical impact: narrow, high-velocity turbulent jets can generate substantial shear stress and hemolysis even when global regurgitant volume appears “moderate,” and eccentric jets may be missed or misgraded unless assessed with a rigorous, integrative approach [1,2].

The reported incidence of PVL varies widely depending on (i) valve position (mitral > aortic in surgical series), (ii) valve type (mechanical > bioprosthetic in many datasets), (iii) imaging intensity (routine TEE vs. symptom-driven evaluation), and (iv) the definition of “clinically significant PVL.” Contemporary reviews and imaging studies frequently cite mitral PVL after surgical replacement in the range of ~7–17%, while clinically significant PVL is much less common (~1–5%) [1,2,4]. In the aortic position after surgical AVR, rates are generally lower; one contemporary cohort study reported postoperative PVL around ~5% overall (with much lower intraoperative PVL), although incidence estimates differ across eras and techniques [5,6]. Long-term surgical data also emphasize that “major” PVL can occur years after implant, reflecting ongoing tissue remodeling, suture degeneration, infection risk, and annular calcification dynamics rather than purely technical failure [7,8,9].

In TAVI, the phenomenon is related primarily to frame–annulus interaction, calcific asymmetry, underexpansion, malposition, and device-generation characteristics. Moderate or severe paravalvular aortic regurgitation has historically occurred in a meaningful minority of early-generation implants and has been repeatedly associated with worse outcomes; while newer devices have reduced rates, PVL remains a persistent “Achilles heel” because even moderate–severe PVL after TAVI is linked to increased mortality across registries and meta-analyses [10,11,12,13].

## 2. Pathophysiology

PVL is fundamentally a mechanical failure of the prosthesis–tissue interface, but its clinical expression is governed by the interaction of (i) the structural substrate (defect geometry, channel length, circumferential extent, and dynamic annular motion), (ii) the fluid mechanics of high-velocity regurgitant flow, and (iii) host factors (tissue quality, infection/inflammation, coagulation/anticoagulation, and red-cell vulnerability). Contemporary reviews emphasize that PVL should be conceptualized less as a single “hole” and more as a spectrum of irregular, often crescentic, multi-orifice channels whose physiology cannot be reliably inferred from a single 2D color Doppler frame [2,14,15].

### 2.1. Structural Substrate

#### 2.1.1. Surgical Prostheses

In surgical valves, PVL most commonly arises from separation of the sewing ring (or annuloplasty ring) from adjacent annular tissue. Early PVL (days–weeks) may reflect technical factors, suture disruption, tension on friable tissue, or unrecognized infection; late PVL (months–years) reflects progressive interface degeneration driven by calcific remodeling, chronic inflammatory processes, and mechanical stress concentration at the ring–tissue boundary. Multiple reviews and consensus endpoint documents consistently list predisposing factors such as annular calcification, tissue friability, prior endocarditis, active corticosteroid therapy, and (in many series) mechanical prosthesis type, particularly in redo settings where annular integrity is compromised [2,14,16,17].

**Figure 1:**
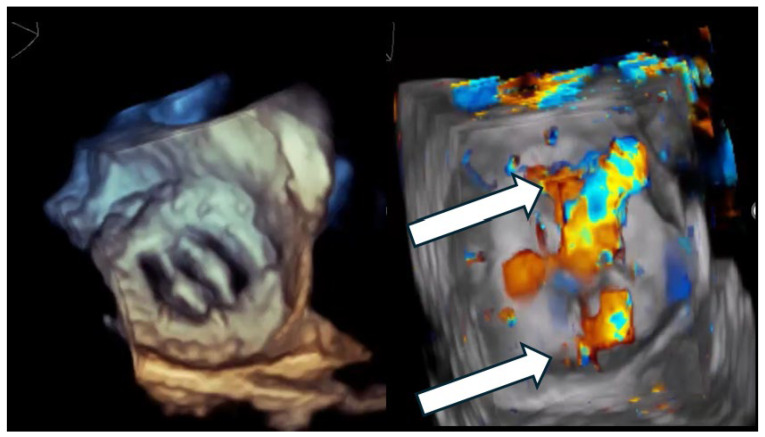
Multi-orifice (two-hole) paravalvular leak demonstrated by three-dimensional transesophageal echocardiography. (**Left panel**): Three-dimensional transesophageal echocardiographic en-face reconstruction of the prosthetic valve illustrating the paravalvular region and surrounding annular anatomy. (**Right panel**): Corresponding three-dimensional color Doppler volume rendering demonstrating two spatially distinct paravalvular regurgitant orifices (arrows) along the prosthetic sewing ring. Each defect generates a separate high-velocity regurgitant jet, confirming the multi-orifice nature of the paravalvular leak rather than a single focal defect.

**Figure 2:**
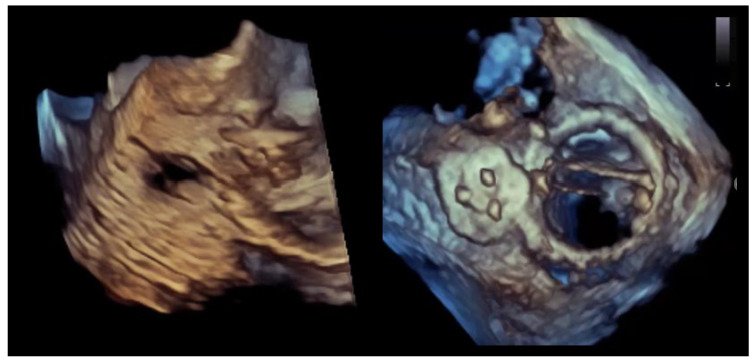
Crescentic paravalvular leak with elongated annular involvement. (**Left panel**): Three-dimensional transesophageal echocardiographic reconstruction of the paravalvular region demonstrating an elongated, crescent-shaped defect extending along the prosthetic sewing ring. (**Right panel**): En-face three-dimensional view illustrating the continuous, non-circular geometry of the paravalvular channel, with multiple potential exit points distributed along the arc of annular dehiscence.

A critical nuance—highly relevant to closure strategy—is that many clinically important PVLs are not circular defects but crescentic arcs along the sewing ring, sometimes with multiple exit points (multiple color jets) from a shared channel (Figure 1 and Figure 2). This morphology explains why “one large round device” frequently fails to eliminate the high-velocity component of flow and why multi-device or oblong/rectangular device concepts have a physiologic rationale [2,14,15].

#### 2.1.2. Transcatheter Valves (TAVI)

In TAVI, PVL (often termed paravalvular regurgitation, PVR) is driven less by “dehiscence” and more by incomplete sealing between the stent frame and the native annulus/leaflet–calcification complex. Mechanistically, PVR reflects a combination of annular eccentricity, asymmetric or bulky calcification at the landing zone, prosthesis malposition (too high/too deep), and underexpansion. Importantly, PVR after TAVI often consists of multiple, eccentric, irregular jets that follow serpiginous channels along calcific shelves—morphology that differs from classic surgical PVL and complicates both quantification and closure [18,19,20]. Contemporary transcatheter valve prostheses incorporate an external prosthetic sealing skirt designed to enhance annular apposition and reduce PVR by sealing small residual gaps at the prosthesis–tissue interface.

Calcification burden and distribution are particularly influential: studies linking device-landing-zone calcification to residual PVL provide a direct structural explanation for why some anatomies remain challenging despite the presence of contemporary prosthetic sealing skirts [18,20].

### 2.2. Fluid Mechanics

PVL flow typically traverses a short, constricted channel with a high-pressure gradient (especially left-sided lesions), producing high-velocity turbulent jets. Turbulence and abrupt changes in flow direction generate regions of elevated shear stress, flow separation, and vortical structures—features that (i) amplify energy loss and hemodynamic burden and (ii) drive hemolysis when shear exposure exceeds red-cell tolerance. Large outcome series and interventional reviews explicitly attribute PVL-related hemolysis to turbulent flow through the defect increasing red blood cell shear stress, causing mechanical trauma and fragmentation [14,16].

Computational fluid dynamics (CFD) work underscores a key clinical observation: hemolysis severity does not scale linearly with a single geometric parameter (e.g., “defect diameter”). Instead, hemolysis risk relates to where shear concentrates, how long erythrocytes are exposed, and what volume of blood experiences suprathreshold shear. In a CFD simulation study of mitral PVL, investigators reported no simple relationship between PVL geometry and hemolysis risk; rather, hemolytic potential was associated with the millisecond-scale exposure time of red blood cells to shear stresses exceeding critical thresholds and the volume of flow experiencing such conditions, with clinically relevant hemolysis arising from the cumulative effect of repetitive exposure over many cardiac cycles [21,22].

This explains why a “moderate” PVL by regurgitant fraction can still cause severe hemolysis (if a narrow, high-velocity component persists), and reducing PVL from moderate to mild may improve HF symptoms, yet hemolysis may persist unless the high-shear micro-jet is eliminated.

### 2.3. Chamber-Specific Hemodynamic Consequences

#### 2.3.1. Mitral PVL

Mitral PVL behaves physiologically like eccentric MR but with important differences: jets are frequently highly directional, may hug the atrial wall, and can be underestimated by transthoracic imaging. The immediate hemodynamic signature in significant mitral PVL is elevation in left atrial pressure and V-wave, with secondary pulmonary venous hypertension and downstream RV strain. Chronicity drives LA remodeling, atrial arrhythmias, and progressive pulmonary vascular disease in susceptible patients. Reviews emphasize that mitral PVL commonly presents with HF symptoms and may coexist with hemolysis, particularly in mechanical valves where hinge-adjacent jets are especially shear-intensive [2,14,15].

#### 2.3.2. Aortic PVL

Aortic PVL creates diastolic runoff from the aorta into the LV, reducing aortic diastolic pressure and increasing LV end-diastolic volume. Clinically, this translates into exertional dyspnea, reduced forward cardiac output reserve, and in severe cases, hypotension or angina (via reduced coronary perfusion pressure). In TAVI specifically, multiple analyses have shown that moderate–severe PVR is prognostically adverse, supporting the concept that even “modest” residual PVL can be physiologically consequential in a population with limited reserve [19,20].

#### 2.3.3. Tricuspid and Pulmonary PVL

Right-sided PVLs are rarer but illustrate an important physiologic contrast. Lower trans-lesional pressure gradients are generally associated with lower jet velocities and, consequently, a reduced propensity for shear-mediated hemolysis; however, gradient alone does not determine hemolysis risk. Hemolysis may still occur in the presence of narrow or highly restrictive paravalvular channels, even at modest pressure differentials. In contrast, volume loading of the right atrium and right ventricle may produce profound systemic venous congestion, including ascites, hepatic congestion, and cardiorenal syndrome. The low-pressure environment may also reduce prosthesis–tissue “self-anchoring” forces, increasing the risk of occluder instability or embolization and making stability testing physiologically—not merely technically—essential [14,15].

### 2.4. Hemolysis

Across modern surgical and transcatheter prostheses, “subclinical hemolysis” can be seen, but clinically relevant hemolysis is most strongly linked to high-shear lesions, especially PVL. A contemporary JACC-focused review on mechanical hemolysis in valvular disease synthesizes how shear stress from turbulent jets—before and after valve interventions—drives red-cell deformation, membrane fatigue, and fragmentation, and it emphasizes that anemia severity is modulated by baseline hematologic reserve and iron handling [16,23].

Experimental and modeling literature has proposed shear thresholds above which hemolysis becomes likely. In this literature, shear stress is expressed in Pascals (Pa; 1 Pa = 1 N/m^2^), and in vitro and computational fluid dynamics models frequently reference values on the order of ~300 Pa as being associated with erythrocyte injury. Importantly, these values represent mechanistic reference points derived from experimental systems, and hemolysis is recognized to depend on both shear magnitude and erythrocyte exposure time, rather than on any single numerical threshold [21,24,25].

The practical clinical implication is that hemolysis is often a “micro-jet disease,” not a “regurgitant volume disease.” Thus, in hemolysis-predominant PVL, the procedural endpoint should be framed as elimination of the high-velocity residual component, not merely downgrading severity by an integrative qualitative grade.

From a mechanistic standpoint, hemolysis severity tends to increase when: (i) the PVL channel is narrow with a steep pressure gradient (high velocity), (ii) there is impingement of the jet on prosthetic struts/hinges (increasing turbulence), (iii) the channel has complex curvature producing local shear hot-spots, and (iv) host factors reduce red-cell resilience (iron deficiency, renal dysfunction, inflammatory states) [21,22,25].

Outcome registries have shown that patients presenting with hemolysis—especially with mechanical valves—have a lower likelihood of meeting composite “clinical success” definitions after transcatheter PVL closure. The most physiologically plausible explanation is that even mild residual PVL can maintain a high-shear micro-jet sufficient for ongoing hemolysis, whereas HF symptoms may improve with partial reduction [26].

### 2.5. Infection and Inflammation

PVL can be both a consequence of infective endocarditis (IE) via annular abscess, tissue destruction, and dehiscence; and a substrate for IE by creating areas of abnormal flow, endothelial disruption, and potential microthrombus formation.

Major PVL reviews emphasize the association between clinically significant PVL and IE, particularly in cases of rapid PVL progression, new dehiscence, systemic inflammatory features, or imaging signs of periannular complications [2,15].

Mechanistically, ongoing inflammatory or degenerative processes at the annulus can lead to progressive separation over time, explaining late PVL and the not-uncommon need for repeat intervention when the underlying interface remains biologically unstable.

### 2.6. Integrative Pathophysiology

Putting the above together, PVL physiology naturally clusters into two dominant, sometimes overlapping phenotypes: (i) HF phenotype: driven by regurgitant volume and chamber pressure/volume loading (LA pressure and pulmonary venous hypertension in mitral PVL; diastolic runoff and LV volume load in aortic PVL), and (ii) Hemolysis phenotype: driven by high-shear turbulent micro-jets through constricted channels, often amplified by jet impingement on prosthetic structures.

This phenotype axis is not merely descriptive—it dictates which imaging features matter most (circumferential extent/volume load vs. micro-jet localization), what procedural endpoint is truly “physiologic success,” and why multi-device strategies are often necessary even when the residual grade appears “acceptable” by conventional echo descriptors.

## 3. Severity Assessment

Unlike native-valve regurgitation, PVL is typically eccentric, frequently multi-orifice, often crescentic, and commonly affected by acoustic shadowing from the prosthesis and surrounding calcification. Single-parameter approaches—particularly reliance on a single 2D VC width—are generally unreliable in PVL assessment. This limitation reflects the typical PVL morphology, which is frequently eccentric, crescentic, multi-orifice, and affected by acoustic shadowing from prosthetic material. Consequently, VC width should not be used as a standalone quantitative measure of PVL severity, especially in the mitral position or when a single dominant jet cannot be clearly isolated. Contemporary prosthetic valve imaging guidance therefore emphasizes multiview, multiparametric echocardiography complemented by CT for anatomic definition and CMR when quantification remains uncertain or when echo/angiography are discordant [27,28,29,30].

A second, clinically crucial nuance is that PVL “severity” has two partially dissociable dimensions: (i) global regurgitant burden (driving HF physiology), and (ii) localized high-shear jet physiology (driving hemolysis). This is why “mild residual PVL” may be clinically acceptable in an HF phenotype yet insufficient in hemolysis-dominant PVL, where the procedural target becomes elimination of the high-velocity residual micro-jet rather than merely downgrading an overall grade [17,26].

Historically, many studies reported PVL in 3 classes (mild/moderate/severe). However, intermediate grades are common and difficult to estimate reproducibly—particularly after TAVI, where multiple eccentric jets are typical. Both VARC-3 and the PVL Academic Research Consortium recommend a 5-class scheme (trace; mild; mild–moderate; moderate; moderate–severe; severe) to improve communication, trial standardization, and prognostic discrimination [17,31,32].

### 3.1. Mitral PVL

For mitral PVL, TEE—particularly 3D TEE—is usually central because of proximity and superior paravalvular delineation compared with TTE. 3D color Doppler enables en-face “surgeon’s view” mapping of PVL location, circumferential extent, and (when feasible) planimetry of the regurgitant orifice/channel entrance, which is often crescentic rather than circular [28,30,33,34].

Core qualitative/semiquantitative markers include (i) jet number and distribution: multiple jets may reflect a single crescentic channel with multiple exits, (ii) proximal jet width/circumferential extent along the sewing ring (short-axis or en-face 3D), (iii) CW Doppler density and contour (supportive only), (iv) pulmonary venous flow: systolic blunting or reversal supports significant MR physiology (interpret with AF, elevated LA pressure, and mitral stenosis cautiously), and (v) LA pressure surrogates: large V-waves (invasive or echo surrogate) support hemodynamic significance.

Classical PISA/EROA methods are frequently unreliable in PVL (non-hemispheric convergence, multiple orifices, irregular channels). Volumetric regurgitant calculations may be attempted but are error-prone in AF or when coexistent lesions exist. Consequently, guidelines endorse an integrated approach and recommend adjunct imaging when clinical severity and echo grade are discordant [27,28,30].

### 3.2. Aortic PVL

In the aortic position, TTE is often more informative than TEE for Doppler quantification, but TEE and especially 3D TEE can be valuable for mechanism and jet localization (particularly intraprocedurally) [28,30]. Echo parameters used in an integrative grading strategy include: (i) circumferential extent of paravalvular jets in short-axis (key discriminator in many schemas), (ii) VC width, or summation of VC widths across multiple jets, used only as a supportive descriptor and recognizing important limitations related to acoustic shadowing, eccentricity, and multi-jet anatomy, (iii) diastolic flow reversal in descending/abdominal aorta (holodiastolic reversal supports severe AR physiology but depends on HR, compliance, and BP), (iv) pressure half-time (supportive but confounded by LV compliance/acute hemodynamics). Several integrative schemas relate circumferential jet extent to severity strata (commonly around the 10–20–30% landmarks) and use these as anchors for intermediate grades in expanded grading systems, recognizing that precision is imperfect and should be corroborated by other hemodynamic markers [31,32,35].

### 3.3. Multimodality Imaging

#### 3.3.1. Cardiac CT

CT is less focused on grading regurgitant severity and more on defining the anatomic substrate of PVL. Using ECG-gated, contrast-enhanced acquisitions with multiplanar and en-face reconstructions, CT is able to delineate the spatial extent of the paravalvular channel or dehiscence along the prosthesis–annulus interface, including channel continuity and arc length, as well as its relationship to prosthetic struts or leaflets, surrounding calcification, coronary ostia, and adjacent structures. Importantly, CT-derived PVL “length” represents an anatomic descriptor inferred from contrast-filled separation planes or crescentic gaps rather than a flow-based measurement, and it is primarily relevant for procedural planning—such as device selection, orientation, anticipated need for multi-device strategies, and fluoroscopic projection planning—rather than as a surrogate of hemodynamic severity. Accordingly, PVL length assessed by CT should be interpreted in conjunction with echocardiographic and clinical data. Current guidelines increasingly recognize the complementary role of CT in prosthetic valve dysfunction assessment, particularly when echocardiographic windows are limited or acoustic shadowing obscures defect morphology [28,30].

#### 3.3.2. CMR

CMR phase-contrast flow imaging can provide robust quantification of aortic regurgitation burden when echo/angiography are discordant, and multiple studies demonstrate that echo can underestimate post-TAVI regurgitation compared with MRI/CMR [36,37,38]. A clinically useful and widely cited post-TAVI CMR approach is regurgitant fraction (RF) grading. In a multicenter study, CMR AR grades were defined as none/trace RF < 15%, mild 16–29%, and moderate/severe ≥ 30%, and RF (per 5% increase) was strongly associated with mortality and HF rehospitalization [38]. This supports a pragmatic paradigm: use echo for mechanism + localization + bedside decisions, and use CMR for definitive regurgitant burden when needed for adjudication, prognostication, or unexplained symptoms.

#### 3.3.3. Angiography

Aortic root angiography (often Sellers’ visual grading) is widely used immediately after TAVI because it is quick and “minimalist,” but it is operator dependent and shows only modest agreement with echo parameters; overlap between grades is substantial [39,40]. Novel quantitative aortography/videodensitometry approaches have been proposed to improve objectivity, but availability and workflow integration vary across centers [41].

## 4. Procedural Endpoints for PVL Closure

PVL closure is prone to heterogeneity in reporting because success can be expressed as: (i) final residual PVL severity (“≤mild”), (ii) relative reduction (“≥1 grade reduction”), or (iii) clinical response (NYHA improvement; transfusion independence).

To address this, the PVL Academic Research Consortium proposed standardized clinical trial principles and endpoint definitions, including a consistent severity scheme and discrete procedural success categories [17]. Similarly, post-TAVI device success and PVR reporting are framed within VARC-3 definitions [31].

### 4.1. Technical Success

Across the PVL literature and consensus documents, “technical success” generally includes (i) successful delivery and stable deployment of closure device(s) across the intended PVL channel(s), (ii) no interference with prosthetic leaflet/disc motion or prosthesis function, (iii) no need for emergency surgery because of device-related complications, and (iv) no intraprocedural catastrophic events (often including death/stroke in ARC-style definitions) [17].

### 4.2. Procedural Success

Many registries define procedural success as technical success plus a meaningful reduction in PVL (commonly ≥ 1 grade reduction or reduction to no more than mild/moderate, depending on the grading system used) [1,2,42]. Because grading schemas differ (3-class vs. 5-class), the manuscript should state the exact scale and the target residual grade a priori.

### 4.3. Clinical Success

Clinical success should be explicitly tied to the presenting phenotype:

- HF phenotype: improvement by ≥1 NYHA class, improved functional capacity, and/or reduction in HF hospitalizations;
- Hemolysis phenotype: improvement in hemolysis markers and, critically, freedom from transfusion/erythropoietin dependence, when hemolysis is the dominant indication.

This definition is consistent with widely cited outcome studies and remains pragmatic for real-world registries. Notably, multicenter data suggest lower clinical success among patients whose index phenotype is hemolysis and/or in mechanical valves—supporting the mechanistic concept that even “small residual PVL” can maintain high-shear hemolysis [17,26,43]. Table 1 summarizes the phenotype-driven severity anchors and procedural endpoints for PVL closure.

## 5. Devices for Transcatheter PVL Closure

PVL channels are rarely circular; they are frequently crescentic, multi-orifice, and short, irregular tunnels between the sewing ring/stent frame and annular tissue. As a result, the “ideal” PVL occluder should (i) conform to noncylindrical geometry, (ii) provide high sealing efficiency (often with fabric), (iii) remain repositionable/retrievable until final leaflet-motion and residual-jet checks are complete, and (iv) minimize leaflet/disc interference and LVOT/coronary impingement. The limitations of using non-dedicated vascular plugs for PVL—especially the risk of incomplete sealing and leaflet interaction—have been demonstrated in bench and clinical literature and directly motivated dedicated PVL platforms [1,44,45].

### 5.1. Dedicated PVL Occluders

#### 5.1.1. Occlutech Paravalvular Leak Device (PLD)

**Figure 3:**
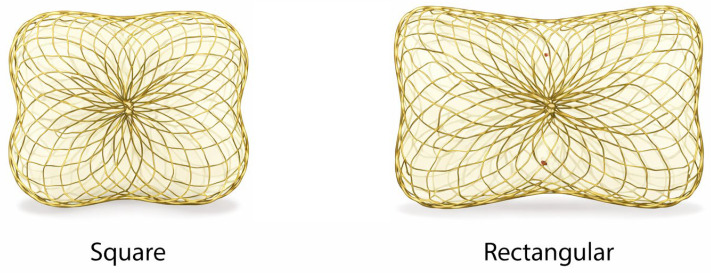
Occlutech Paravalvular Leak Device (PLD). Representative image of the Occlutech paravalvular leak device illustrating its square and rectangular configurations. The device consists of a self-expanding nitinol mesh with integrated polyethylene terephthalate (PET) fabric patches designed to promote local thrombosis and enhance sealing of crescentic or irregular paravalvular channels.

The Occlutech PLD (Figure 3) is a double-disc nitinol device engineered specifically for PVL anatomy, offered in square and rectangular configurations to better match crescentic/elliptical channels. The device incorporates polyethylene terephthalate (PET) fabric patches within the nitinol framework, which promote local thrombosis and tissue ingrowth, thereby enhancing sealing efficiency of the paravalvular channel and reducing residual regurgitant flow. It is repositionable and fully retrievable and is designed for delivery via antegrade (e.g., transseptal) or retrograde routes depending on anatomy and operator strategy [46,47]. Large multicenter experience has shown high implant and clinical improvement rates with the PLD in both mitral and aortic PVL [46,47]. Longer-term follow-up demonstrated sustained improvement in NYHA class and reduced transfusion dependence in hemolysis-driven cases, with no deaths adjudicated as device-related [48].

Device selection “pearls” (device-specific).

- Rectangular PLD: best suited for elongated/crescentic defects where sealing is required along an arc;
- Square PLD: useful for more compact defects;
- Channel characteristics matter: registry/experience papers stress matching device configuration to channel length and cross-sectional area, avoiding inappropriate oversizing that can increase interference risk [46,47].

#### 5.1.2. Amplatzer Vascular Plug (AVP)

**Figure 4:**
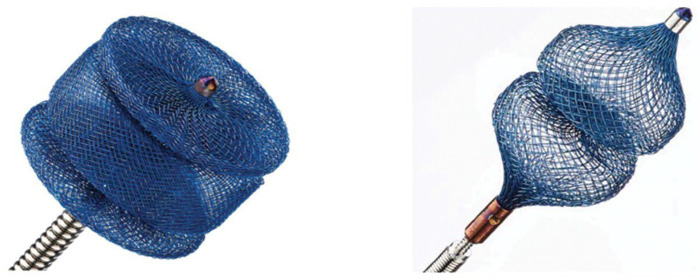
Amplatzer Vascular Plug (AVP). Representative image of the Amplatzer Vascular Plug paravalvular leak devices. Reproduced from Ref. [54].

The Amplatzer plug family (Figure 4) remains the most widely used platform for transcatheter PVL closure in routine practice, spanning earlier cylindrical vascular plugs (AVP II), low-profile options (AVP IV), and the oblong/rectangular-oval Amplatzer Valvular Plug III (historically reported in several series as “AVP III”), which was developed to better match the common elliptical/crescentic morphology of PVL channels and reduce “round-hole mismatch,” while maintaining a self-expanding nitinol architecture and retrievability/repositionability—features that are particularly valuable when defects are adjacent to mechanical hinges or transcatheter valve frames [49,50]. Among the largest early clinical experiences, mitral/aortic PVL closure with AVP III achieved high implant success and clinically meaningful PVL reduction, while also underscoring a key device-selection constraint: prosthetic leaflet interference, though infrequent, can be catastrophic and should be actively excluded before release [49,51]. Prospective evidence generation is ongoing through the PARADIGM multicenter single-arm study evaluating safety and effectiveness of AVP III in clinically significant aortic/mitral PVL after surgical valve implantation [52]. A single-center “rectangular PVL plug” experience reported favorable mechanical behavior (auto-orientation, minimal leaflet interaction in that cohort) but also highlighted the practical reality that crescentic PVLs frequently require multi-device strategies even with an oblong platform [53]. In contrast, AVP II remains useful primarily for more tubular/round channels where a cylindrical plug can seat securely, but experimental in vitro work demonstrated that vascular plugs may not reliably produce substantial PVL reduction and can interact with prosthetic leaflets—supporting the preference for morphology-matched devices and careful intraprocedural imaging checks [26,44]. Finally, AVP IV has become a practical “workhorse” in anatomies where crossability and deliverability are limiting—particularly post-TAVI PVL constrained by stent-frame geometry—an observation reflected in post-TAVI PVL literature and in the international PLUGinTAVI registry, where AVP III was most frequently used, followed by AVP IV [10].

### 5.2. Ductal, Septal, and VSD Occluders

These devices predate dedicated PVL platforms and were historically used because PVL-specific devices were not available. Contemporary series still employ them as “niche tools,” particularly when a PVL channel behaves like a short duct/tunnel and disc-based anchoring is advantageous.

#### 5.2.1. Amplatzer Duct Occluder (ADO) and ADO II

Disc-based occluders can be useful when a PVL channel has a tubular segment that can seat the waist while the discs provide stability. ADO II has been reported for mitral PVL closure (including retrograde approaches in select cases) and has also been described in tricuspid PVL closure in high-risk surgical patients [55,56].

Because ADO II is designed with two articulating discs and a conformable mesh architecture, it may be deliverable through relatively low-profile systems (depending on size), which can be a practical advantage in difficult-to-cross channels [56].

#### 5.2.2. Muscular VSD Occluders and ASD Occluders

Muscular VSD occluders offer larger discs and waist configurations that can be helpful for larger PVL channels with adequate landing zones; ASD occluders have also been used historically. However, larger discs can increase the risk of prosthetic leaflet/disc interference—a complication repeatedly observed across PVL closure experience and one of the core reasons dedicated PVL devices with optimized profiles were developed [49,57,58].

### 5.3. Coils and Adjunctive “Micro-Jet” Solutions

For very small residual PVL jets (particularly when hemolysis persists from a high-shear residual micro-jet), coils have occasionally been used as adjuncts in combination with plugs, but their role is limited by the need for stable anchoring, embolization risk, and less predictable sealing compared with fabric-containing occluders. Most contemporary practice patterns favor plug-based solutions for reproducibility and retrievability, reserving coils for exceptional anatomies or residual pinhole channels once a stable scaffold is established.

### 5.4. Multi-Device Strategies and Device Combinations

A critical device-related principle is that many clinically significant PVLs—especially crescentic defects spanning a substantial arc—are unlikely to be fully sealed with a single plug. Classic interventional reviews state that crescent-shaped defects extending over a substantial portion of the prosthesis circumference often require two or more devices, deployed sequentially or simultaneously, to achieve near-complete sealing [58,59].

This is particularly relevant to hemolysis phenotypes, where leaving even a small residual high-velocity component can perpetuate hemolysis.

In real-world series, even when a dedicated oblong/rectangular plug is used, additional devices may be required to address multi-orifice exits or to “finish” residual channels—an observation explicitly reported in rectangular PVL plug experience [53].

Table 2 summarizes the characteristics of different PVL closure device platforms

## 6. Procedural Workflow for Transcatheter PVL Closure

**Figure 5:**
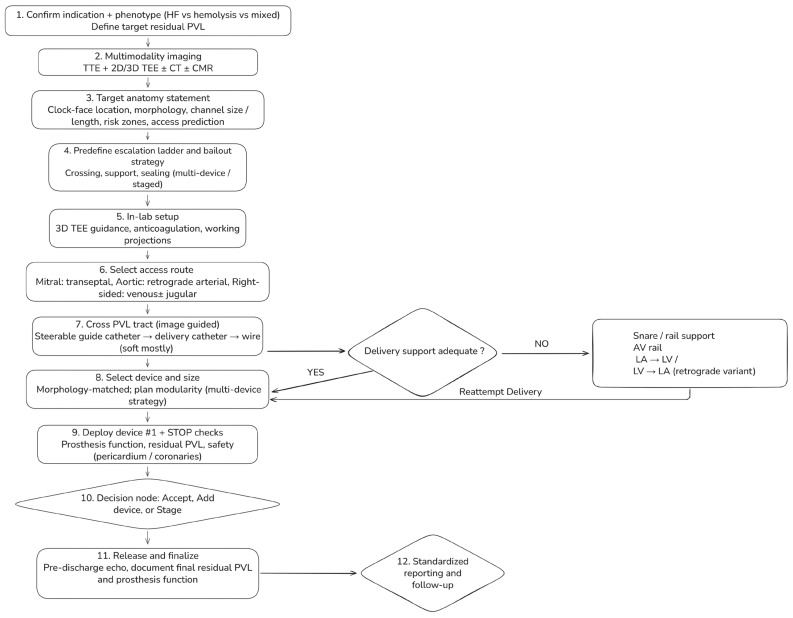
Step-by-step workflow for transcatheter paravalvular leak (PVL) closure. A standardized, phenotype-driven workflow integrating multimodality imaging, access planning, and planned escalation strategies. The process begins with confirmation of indication and dominant phenotype (heart failure, hemolysis, or mixed), which defines the target residual PVL. Pre-procedural assessment combines transthoracic and 2D/3D transesophageal echocardiography with selective use of cardiac CT and CMR to generate a target anatomy statement. In-lab steps include access selection according to valve position, image-guided PVL crossing, and evaluation of delivery support. When support is inadequate, snare-assisted or arteriovenous rail techniques are used before reattempting delivery. Devices are selected according to defect morphology, with modular multi-device strategies for complex channels. Mandatory STOP checks precede device release, followed by a decision to accept, add devices, or stage the procedure, and standardized post-procedural reporting and follow-up.

Transcatheter paravalvular leak (PVL) closure should follow a standardized, phenotype-driven workflow integrating multimodality imaging, access planning, and predefined escalation strategies (Figure 5). The process begins with confirmation of the indication for intervention and identification of the dominant clinical phenotype—heart failure, hemolysis, or mixed—which together define the target residual PVL.

Pre-procedural assessment combines transthoracic and two- and three-dimensional transesophageal echocardiography, with selective use of cardiac CT or CMR when echocardiographic findings are discordant, anatomy is complex, or cross-sectional definition is required. Before entering the catheterization laboratory, the Heart Team should agree on a concise target anatomy statement summarizing valve position and prosthesis type, PVL location using standardized clock-face orientation, defect morphology (focal vs. crescentic or multi-orifice), estimated channel dimensions, and relevant anatomic risk zones.

Intraprocedurally, access strategy is selected according to valve position, followed by image-guided PVL tract crossing and early escalation to enhanced support techniques when deliverability is limited. Device selection is guided primarily by defect morphology, with planned modular or multi-device strategies favored for crescentic or complex channels. Mandatory safety checks precede device release, followed by a structured decision to accept the result, escalate with additional devices, or stage the procedure. Standardized post-procedural reporting and phenotype-linked follow-up complete the workflow.

Detailed technical maneuvers, escalation algorithms, and troubleshooting strategies are provided in the Supplementary Materials.

## 7. Valve-Specific Considerations

### 7.1. Mitral PVL

Mitral PVL is often the most technically demanding subtype because transseptal trajectories are frequently non-coaxial, defects are commonly crescentic with multi-orifice exits, and prosthesis-related shadowing may obscure residual jets. Contemporary practice therefore emphasizes three-dimensional TEE en-face mapping for localization and intraprocedural guidance, with selective CT use in complex redo anatomies. Because crescentic defects are common, single-device closure is frequently insufficient, and morphology-matched, modular multi-device strategies are often required, particularly in hemolysis-predominant presentations. In mechanical mitral prostheses, any leaflet or hinge interaction mandates immediate recapture and strategy revision.

### 7.2. Aortic PVL

Surgical aortic PVLs are often more focal and amenable to retrograde arterial closure. The principal intraprocedural hazards are prosthetic leaflet restriction and coronary compromise; imaging strategy and projection selection should therefore prioritize clear separation between the PVL channel, prosthetic struts, and coronary ostia. When echocardiographic and angiographic grading are discordant, adjunctive hemodynamic assessment or follow-up quantification may be required.

### 7.3. Post-TAVI PVR

Post-TAVI PVL is mechanistically distinct, characterized by eccentric multi-jet flow along calcific shelves and valve-frame interfaces. Before plug closure, alternative mechanisms such as prosthesis underexpansion, malposition, or predominant transvalvular regurgitation should be systematically excluded. Deliverability frequently dictates device choice, with low-profile platforms favored in narrow or angulated channels.

### 7.4. Right-Sided PVLs

Right-sided PVLs are uncommon but highlight an important physiologic contrast. Lower pressure gradients are generally associated with a lower propensity for shear-mediated hemolysis, whereas low-pressure physiology increases the risk of device instability and embolization. Accordingly, physiologic stability testing before release is essential, and anchoring considerations often outweigh aggressive sealing.

## 8. Special Scenarios

### 8.1. Active or Recent Infective Endocarditis

Active infective endocarditis remains a contraindication to transcatheter PVL closure in nearly all cases because of ongoing tissue destruction and device instability. Catheter-based therapy may be considered only exceptionally, after infection control, in prohibitive surgical-risk patients with well-defined and stable anatomy.

### 8.2. Large Circumferential Dehiscence (“Rocking Prosthesis”)

Extensive dehiscence with prosthetic instability is primarily a surgical problem. In carefully selected non-operative patients, limited case-based experience suggests that modular percutaneous strategies may provide palliation, but only with strict imaging safeguards and a low threshold for procedural termination.

### 8.3. Crescentic, Multi-Orifice PVLs and Hemolysis-Dominant Phenotypes

Crescentic or multi-orifice PVLs frequently fail single-device closure. In hemolysis-dominant cases, even small residual high-velocity micro-jets may perpetuate clinical failure; escalation to additional devices should be considered when prosthetic function and stability are preserved.

A detailed tabulated overview of special scenarios, decision points, and procedural hazards is provided in the Supplementary Materials.

## 9. Outcome Evidence

The contemporary outcomes literature for transcatheter paravalvular leak (PVL) closure is largely derived from observational cohorts, multicenter registries, and meta-analyses of non-randomized data. Interpretation is limited by substantial heterogeneity in: (i) baseline patient risk (frequently prohibitive risk for redo surgery), (ii) PVL substrate and mechanism (e.g., suture dehiscence, annular calcification–related channels, endocarditis-related pathology), (iii) prosthesis characteristics (mechanical vs. bioprosthetic; surgical vs. transcatheter), and (iv) endpoint definitions (with variable and sometimes non-comparable definitions of technical, procedural, and clinical success). To address these limitations, the PVL Academic Research Consortium proposed core methodological principles and standardized endpoint definitions for PVL studies, including a structured PVL grading scheme and clinically meaningful composite outcomes integrating hemodynamic/imaging metrics with patient-centered endpoints [17].

Across essentially all available datasets, the most reproducible treatment-response signal is determined by residual PVL severity, rather than device deployment per se. Procedural efficacy is therefore best conceptualized not as “device implanted,” but as achievement of ≤mild residual regurgitation without prosthetic leaflet impairment, device embolization, or major periprocedural complications. Persistent ≥moderate residual PVL is consistently associated with adverse clinical trajectories, including higher mortality, increased rehospitalization and reintervention rates, and persistence of hemolysis and/or heart failure. This concept is embedded in ARC-style endpoint frameworks and is repeatedly corroborated in single-center and multicenter outcomes analyses [17,60].

A contemporary benchmark is provided by a prospective, international European multicenter registry (FFPP; 24 centers, 2017–2019) enrolling 216 symptomatic patients undergoing 238 PVL closure procedures (mitral 64.3%, aortic 34.0%, tricuspid 1.7%), with heart-failure–predominant, hemolysis-predominant, and mixed phenotypes in 48.9%, 7.8%, and 43.3%, respectively. The registry reported successful implantation with PVL reduction to ≤grade 2 in 85.0% of mitral and 91.4% of aortic procedures, with low major periprocedural adverse event rates (3.3% mitral; 1.2% aortic). Importantly, a stringent 1-month clinical success composite (absence of heart-failure readmission, transfusion, surgery, or death) differed by valve position (70.3% for mitral vs. 88.0% for aortic PVL closure), underscoring that similar device-implant success does not necessarily translate into equivalent short-term clinical benefit across anatomies and phenotypes [26]. The FFPP registry also provides multivariable data highlighting mechanisms of clinical non-response despite an apparently satisfactory intraprocedural result: independent correlates of failure to achieve clinical success included technical failure (OR 7.7), mechanical prosthesis (OR 3.6), and a hemolytic anemia phenotype (OR 3.7).

In parallel, structured single-center “real-world” series with longitudinal follow-up commonly report technical success near ~90% and procedural success near ~75–80%, reflecting frequent use of multiple devices and staged strategies in complex, crescentic, channel-like defects. For example, one long-term single-center experience of percutaneous mitral PVL closure reported technical success of 89.8% and procedural success of 79.2%, with >1 device used in 32% and planned staged procedures in 16.7%, aligning with expected requirements for modular sealing strategies in elongated crescentic tracks [1].

In the absence of randomized trials, one of the strongest inference signals comes from analyses stratifying outcomes by successful versus unsuccessful PVL reduction. A Bayesian hierarchical meta-analysis (12 studies; 362 patients) reported that successful transcatheter PVL reduction was associated with lower cardiac mortality (OR 0.08, 95% credible interval 0.01–0.90), substantially higher odds of clinical improvement in NYHA class and/or hemolysis parameters (OR 9.95, 95% CrI 2.10–66.73), and fewer repeat surgical interventions (OR 0.08, 95% CrI 0.01–0.40) [61].

Large single-center cohorts further support this relationship using clinically interpretable event rates. In a Mayo Clinic cohort of 231 percutaneous mitral PVL repairs (2006–2017), 70% achieved ≤mild residual PVL. Relative to patients with >mild residual PVL, achievement of ≤mild residual PVL was associated with lower repeat surgical intervention (6% vs. 17%), lower 30-day mortality (1% vs. 14%), lower 1-year mortality (15% vs. 39%), and improved 3-year survival (61% vs. 47%) [60]. These findings operationalize procedural endpoint quality into tangible differences in survival and reintervention and support the clinical implication that residual ≥moderate PVL is rarely an acceptable endpoint in high-risk mitral PVL—particularly in heart failure– and hemolysis-driven phenotypes [60].

More recent long-term analyses consistently employ similar definitions—procedural success as ≤mild residual PVL—and evaluate hard endpoints including all-cause mortality, cardiovascular mortality, and heart failure hospitalization, again reinforcing residual PVL grade as the procedural metric with the greatest prognostic value [62].

Comparative evidence consistently indicates a trade-off: surgery typically achieves higher technical success but at the cost of early hazard and perioperative morbidity, whereas transcatheter closure generally offers a lower early risk profile and faster recovery but with a higher probability of residual PVL and potential need for reintervention. A comprehensive meta-analysis (22 studies; 2373 patients; 63.7% percutaneous) reported higher technical success with surgery (96.7% vs. 72.1%, OR 9.7, *p* < 0.001) but higher 30-day mortality (8.6% vs. 6.8%, OR 1.90, *p* < 0.001), a signal toward higher stroke (3.3% vs. 1.4%), and longer hospitalization. At 1 year, mortality was similar (17.3% vs. 17.2%), and symptomatic improvement did not differ significantly, suggesting that beyond early hazard, the principal differentiator is whether the chosen therapy achieves durable elimination of clinically significant PVL in a given anatomy and clinical substrate [63].

Institutional comparative cohorts complement meta-analyses by providing systems-level endpoints. In one cohort of 114 patients (2007–2016) treated by transcatheter intervention (TI, n = 56) versus surgery (SI, n = 58), TI patients were older and had fewer endocarditis cases; nevertheless, TI was associated with shorter ICU and overall hospital stays and fewer 30-day readmissions, with no significant differences in 1-year survival (83.9% TI vs. 75.9% SI) or the 1-year composite endpoint (death, reintervention, or CHF readmission) [64].

Other long-term comparative analyses suggest that surgery may outperform transcatheter closure for composite long-term endpoints (e.g., death and heart failure hospitalization) in selected cohorts, but often at the cost of substantial perioperative mortality and morbidity. This supports a pragmatic Heart Team synthesis: surgery is generally preferred when feasible and expected to be durable (particularly in the setting of infection or major dehiscence), whereas transcatheter closure is favored when surgical risk is prohibitive or when anatomy is conducive to catheter-based elimination of the dominant culprit jet(s) [61,63]. An open-access meta-analysis (13 studies; 2003 patients) similarly reported lower short-term mortality with transcatheter closure (30-day OR 0.28) while acknowledging the ongoing challenge of residual ≥moderate PVL and persistent symptoms in some datasets—again emphasizing the primacy of endpoint quality (≤mild residual PVL) and careful patient/anatomy selection [65].

Post-TAVI PVL represents a distinct outcomes domain. Anatomy is constrained by the transcatheter frame, calcific shelves, and elliptical/irregular channels; crossing can become the principal rate-limiting step; and procedural success may be constrained by support and deliverability rather than morphology matching alone. The international PLUGinTAVI registry (45 patients across multiple centers) supports feasibility and safety of percutaneous post-TAVI PVL closure and emphasizes that reduction to mild or less is the key determinant of acute and durable clinical improvement in this setting [10].

Overall, contemporary outcomes with modern plug strategies and dedicated PVL devices can be summarized as high implant success in experienced programs, with clinical benefit closely coupled to residual PVL grade and avoidance of device–leaflet interaction. Early foundational experience demonstrated high rates of clinical success among technically successful cases and provided survival benchmarks that established PVL closure as a credible alternative for non-surgical candidates [17]. For the Occlutech Paravalvular Leak Device (PLD), clinical series report high procedural success with signals toward functional improvement and hemoglobin recovery in hemolysis and/or heart failure phenotypes; in one experience (30 patients; 34 PVLs), procedural success was 94.1%, with NYHA class improvement and favorable laboratory trends during follow-up [47].

Quality-of-life (QoL) endpoints remain underreported in structural PVL literature; however, prospective registry data indicate that when procedural success is achieved, QoL improves meaningfully over time across MLHFQ and KCCQ domains, whereas unsuccessful procedures do not demonstrate comparable improvement (noting limited power in failed-procedure subgroups).

## 10. Complications and Bailout Management in Transcatheter PVL Closure

Complications during transcatheter paravalvular leak (PVL) closure are uncommon in experienced centers but can be abrupt and clinically severe when they occur. Importantly, adverse events in this setting most often arise not from device failure per se, but from PVL-specific anatomic constraints, prolonged manipulation along prosthetic structures, and complex support strategies that distinguish these procedures from routine catheterization.

The most consequential PVL-specific complication is prosthetic leaflet or disc interaction, particularly in mechanical valves or crescentic defects with oblique channel orientation [1]. Even intermittent restriction can precipitate acute hemodynamic compromise or severe hemolysis and should be regarded as a high-severity, largely preventable event. Accordingly, preservation of prosthetic valve function represents a non-negotiable procedural endpoint, mandating immediate recapture and strategy revision whenever leaflet motion or transvalvular gradients are affected.

Device embolization reflects inadequate anchoring within short, irregular, or crescentic channels and is more likely in low-pressure right-sided circulations [66,67]. These risks underscore the importance of morphology-matched device selection, planned modular sealing strategies, and rigorous stability confirmation prior to release. Similarly, persistent or worsened hemolysis after partial closure highlights that hemolysis-dominant PVL behaves as a high-shear micro-jet disease; incomplete sealing may paradoxically increase shear stress and perpetuate clinical failure.

Other complications—including thromboembolic events, vascular injury, cardiac perforation, coronary compromise, and conduction disturbances—are influenced by prolonged procedure duration, large-bore access, repeated exchanges, and manipulation near prosthetic or calcified structures [66,67]. Anticipation of these risks, predefined bailout readiness, and Heart Team availability are therefore essential, particularly in complex anatomies.

Detailed mechanisms, preventive strategies, and bailout maneuvers for PVL-specific complications are provided in the Supplementary Materials.

## 11. Conclusions and Future Directions

Transcatheter PVL closure is an imaging-intensive, technically nuanced intervention in which procedural endpoint quality (≤mild residual PVL without prosthetic interaction) is the dominant driver of clinical benefit. A phenotype-informed strategy is essential: HF-predominant cases may improve with meaningful reduction, whereas hemolysis-predominant PVL often requires near-elimination of high-velocity residual jets, frequently via modular multi-device sealing. Optimal outcomes depend on rigorous multimodality characterization of defect geometry and prosthesis relationships, access and support strategies matched to valve position, morphology-adapted device selection, and systematic prevention and management of predictable complications.

Future progress should prioritize: (i) wider adoption of standardized ARC-aligned PVL grading and composite endpoints to improve cross-study comparability; (ii) prospective registries incorporating quality-of-life measures and longer-term durability outcomes; (iii) refinement and broader availability of dedicated PVL devices and delivery platforms; (iv) integration of advanced imaging workflows (CT–TEE fusion, structured 3D quantification, CMR RF-based grading post-TAVI, and selective PET for inflammatory/infective substrates); and (v) pragmatic comparative-effectiveness studies to clarify patient- and anatomy-specific thresholds favoring surgery versus transcatheter therapy. Ultimately, the field’s next step is not simply improving implant success, but reliably achieving durable, hemodynamically meaningful PVL elimination with standardized reporting and patient-centered outcomes.

## Figures and Tables

**Table 1:** Phenotype-driven severity anchors and procedural endpoints for paravalvular leak (PVL) closure.

Predominant Clinical Phenotype	Practical Severity Anchors (Pre-Procedure)	Minimum Imaging Endpoints to Report	Target Residual PVL (Pragmatic)	Clinical Success Endpoint(s)
Heart failure–predominant PVL (mitral)	Symptoms (NYHA); pulmonary venous congestion; LA dilation/V-wave; supportive Doppler signs (e.g., pulmonary venous systolic blunting/reversal when interpretable)	2D/3D TEE mapping (location, circumferential extent, number of jets); pre/post mean mitral gradient; residual PVL grade at exit + predischarge	≤mild–moderate (5-class) or ≤mild (3-class), and no clinically relevant iatrogenic mitral stenosis	≥1 NYHA class improvement; fewer HF admissions; QoL improvement
Heart failure–predominant PVL (aortic/post-TAVI)	Dyspnea/low output; LV volume load; supportive Doppler (diastolic flow reversal)	Echo integrative grade (include circumferential extent); angiography/hemodynamics if used; residual PVL at exit + predischarge	Preferably ≤ mild–moderate; consider hemodynamic optimization during procedure (esp. post-TAVI)	HF rehospitalization reduction; QoL; survival (longer-term)
Hemolysis-predominant PVL (any position; often mechanical)	Hemoglobin trend; LDH ↑; haptoglobin ↓; indirect bilirubin ↑; transfusion/EPO requirement	Precise jet localization (often 3D TEE); document elimination of high-velocity residual micro-jet	As close to none/trace as achievable (hemolysis is “micro-jet sensitive”)	Transfusion independence; improvement/normalization trend of hemolysis labs
Mixed HF + hemolysis	Combined anchors above	Full integrative imaging set + labs	Aim for lowest achievable residual grade without prosthesis compromise	NYHA improvement and hemolysis improvement/transfusion-free

**Table 2:** Device platforms for transcatheter paravalvular leak closure: indications, advantages, and limitations.

Device/Platform	Typical PVL Morphology Where It Fits Best	Key Advantages	Key Limitations/Cautions
Occlutech PLD (square/rectangular; waist/twist)	Crescentic/elliptical PVL; irregular channels (mitral & aortic)	Purpose-built geometry; PET patches; markers; retrievable/repositionable	Requires careful sizing to avoid leaflet interaction; availability varies by region
Amplatzer Valvular Plug III/oblong PVL plug concept	Elliptical/crescentic PVL; multi-orifice channels	Oblong geometry; broad size range; widely used in PVL practice; under formal evaluation (PARADIGM)	Leaflet interference remains a risk; sometimes multiple devices needed
AVP II	More tubular/round PVL channels	Familiar platform; effective occlusion in suitable geometries	Less conformable for crescentic PVL; can interact with leaflets if protruding
AVP IV (low profile)	Small channels; post-TAVI PVL where crossability is limiting	Low-profile deliverability; common in post-TAVI PVL closure practice	Not ideal for large crescentic defects; may require multiple devices
ADO/ADO II	Short tunnel-like PVL; selective mitral/tricuspid PVL cases	Disc-based stability; can be useful when “duct-like” anatomy exists	Off-label in PVL; embolization/interference risk if landing zone is marginal
Muscular VSD/ASD occluders	Large defects with adequate landing zone	Large discs/waist options	Higher interference risk; not designed for PVL geometry

## Data Availability

No new data were created or analyzed in this study.

## Supplementary Materials

The following supporting information can be downloaded at https://www.mdpi.com/article/10.3390/jcdd13020096/s1: Table S1: Valve-specific approach, escalation triggers, and operator pearls for transcatheter PVL closure.; Table S2: Operator troubleshooting guide for PVL closure; Table S3: Special scenarios in transcatheter paravalvular leak (PVL) closure; Table S4: Complication recognition and management during PVL closure.

## Abbreviations

The following abbreviations are used in this manuscript:

2D	Two-dimensional
3D	Three-dimensional
ACT	Activated clotting time
ADO	Amplatzer Duct Occluder
AF	Atrial fibrillation
AR	Aortic regurgitation
ARC	Academic Research Consortium
ASD	Atrial septal defect
ASE	American Society of Echocardiography
AV	Arteriovenous (e.g., AV rail)
AVP	Amplatzer Vascular Plug
AVR	Aortic valve replacement
BP	Blood pressure
CFD	Computational fluid dynamics
CHF	Congestive heart failure
CMR	Cardiac magnetic resonance
CT	Computed tomography
CW	Continuous-wave (Doppler)
EPO	Erythropoietin
ESC	European Society of Cardiology
FFPP	European multicenter PVL registry (Feasibility, First-in-human, and Prospective PVL registry)
HF	Heart failure
HOLE	Spanish HOLE PVL registry
HR	Hazard ratio
ICE	Intracardiac echocardiography
ICU	Intensive care unit
IE	Infective endocarditis
JACC	Journal of the American College of Cardiology
KCCQ	Kansas City Cardiomyopathy Questionnaire
LA	Left atrium
LDH	Lactate dehydrogenase
LV	Left ventricle
LVOT	Left ventricular outflow tract
MAC	Mitral annular calcification
MACE	Major adverse cardiovascular events
MLHFQ	Minnesota Living with Heart Failure Questionnaire
MR	Mitral regurgitation
MRI	Magnetic resonance imaging
NYHA	New York Heart Association (functional class)
OR	Odds ratio
PARADIGM	Multicenter PVL outcomes study (trial acronym)
PET	Polyethylene terephthalate
PISA	Proximal isovelocity surface area
EROA	Effective regurgitant orifice area
PLD	Occlutech Paravalvular Leak Device
PLUGinTAVI	International registry of post-TAVI PVL closure (PLUGinTAVI)
PVL	Paravalvular leak
PVR	Paravalvular regurgitation
QoL	Quality of life
RA	Right atrium
RF	Regurgitant fraction
RV	Right ventricle
RVOT	Right ventricular outflow tract
SI	Surgical intervention
STOP	Standardized pre-release safety checklist (“STOP checks”)
TAVI	Transcatheter aortic valve implantation
TEE	Transesophageal echocardiography
THV	Transcatheter heart valve
TI	Transcatheter intervention
TMVR	Transcatheter mitral valve replacement
TTE	Transthoracic echocardiography
UK	United Kingdom
VARC-3	Valve Academic Research Consortium-3
VC	Vena contracta
VSD	Ventricular septal defect
