# Supplementary material for: Transcatheter Paravalvular Leak Closure: A Step-by-Step Guide
Source: J Cardiovasc Dev Dis. 2026 Feb 16;13(2):96. doi: 10.3390/jcdd13020096 (PMC12941249; doi:10.3390/jcdd13020096)
Supplement: Supplementary file 1 [file jcdd-13-00096-s001.zip › jcdd-4121241-supplementary.pdf]

Supplementary Methods

Table S1. Valve-specific approach, escalation triggers, and operator pearls for transcatheter PVL closure.

Valve position / scenario	Default access	Key imaging guidance	Escalate early when…	Device strategy tendency	Unique hazards / hard-stops
Mitral PVL (surgical MVR / ViV / ViR)	Transseptal	3D TEE en-face ± CT	Prolapse or non-coaxiality → steerable sheath Sheath buckling → AV rail	Crescentic common → oblong/rectangular or multi-device; hemolysis → "micro-jet elimination"	Any hinge or leaflet interaction → immediate recapture
Aortic PVL (surgical AVR)	Retrograde arterial	TTE/TEE integrative + angiography; CT if redo/root complexity	Poor support → long sheath or mother-and-child Multiple jets → reassess anatomy	Often focal → single device	New transvalvular AR or gradient rise → recapture Coronary compromise
Post-TAVI PVR	Retrograde arterial (usually)	TTE/TEE + CT Hemodynamics adjunct	Cannot cross → low-profile devices (e.g. AVP IV class)	Crossability dictates choice Aim ≤ mild residual	Frame interaction Multi-jet eccentricity Angio–echo discordance
Tricuspid PVL	Venous (± jugular)	TEE or ICE	Instability concern → formal stability testing Snare ready	Usually single device Avoid oversizing	High embolization risk → confirm stability across respiration
Pulmonary PVL / RVOT	Venous	CT for conduit geometry	Reach or support limited → long sheaths / rail	Stability-focused	Low-pressure circuit → anchoring must be proven

Table S2. Operator troubleshooting guide for PVL closure

Technical challenge	Recommended approach	Rationale
Cannot engage/cross the PVL channel	Switch fluoroscopic projection; use 3D TEE en-face localization; escalate early to a steerable sheath	Minimizes blind probing; improves coaxial alignment with the paravalvular gutter
Wire repeatedly prolapses	Use a short-tip hydrophilic wire; reduce LV/LA loop	Improves tip control; preserves tract access; reduces traumatic force transmission
Delivery system buckles / cannot advance sheath	Create AV rail (mitral) or selective rail → add long sheath or mother-and-child support	Converts unstable pushing into controlled tracking; stabilizes coaxial delivery
Clinically relevant residual jet persists	Add second device or plan staged modular closure (do not oversize single device)	Crescentic or multi-orifice defects seal more reliably with modular strategy
Any mechanical leaflet/disc interaction	Immediate recapture; downsize and/or reposition; change geometry strategy	Prosthesis safety is non-negotiable; subtle restriction can be catastrophic

Table S3. Special scenarios in transcatheter paravalvular leak (PVL) closure

Special scenario	Why it is different	Preferred strategy	Red flags	Imaging focus
Active infective endocarditis	Ongoing tissue destruction; unstable borders; embolic risk	Defer PVL closure; Heart Team–led IE management; consider catheter therapy only as exceptional palliation after infection control	Persistent bacteremia/fever; abscess, pseudoaneurysm, fistula → usually surgical	TEE for dehiscence/abscess; CT/FDG-PET as indicated
Healed IE with pseudoaneurysm or fistula	Acts as tract communication, not simple PVL	Treat as structural tract closure; define entry/exit; staged or modular strategy	Uncertain infection status; expanding pseudoaneurysm; poor landing zone	TEE + CT to define tract, neck, coronary/valve relation
Large circumferential dehiscence ("rocking" valve)	Valve instability dominates, not discrete channel	Surgical evaluation first; percutaneous only in prohibitive risk with strict stop criteria	>⅓ circumference dehiscence; severe rocking; unstable borders	3D TEE for extent/motion; CT for annular integrity
Multiple PVLs or crescentic multi-orifice channel	Single-plug strategy often fails	Planned multi-device closure; scaffold first, then remap and seal residual exits	Oversizing causing hinge interaction; accepting residual micro-jet in hemolysis	3D TEE en-face + residual mapping; CT for complex tracts
Hemolysis-dominant PVL	High-shear micro-jets drive clinical failure	Endpoint = eliminate high-velocity jet; low threshold for second device; track labs	Persistent micro-jet; leaflet restriction; unstable seal	3D TEE jet localization; standardized hemolysis labs
Mechanical prosthesis	Catastrophic failure if leaflet/disc restricted	Strict release discipline; prefer low-profile, modular strategy	Any leaflet restriction, gradient rise, or new transvalvular regurgitation	Continuous leaflet motion + gradients during positioning
Post-TAVI PVL (PVR)	Multi-jet eccentric flow; frame/calcification constraints	Optimize mechanism first (post-dilation/ViV); low-profile crossing; aim ≤ mild	Predominant transvalvular AR; unstable valve position; cannot cross safely	VARC-aligned echo; CT for frame/trajectory/coronaries
Combined valve therapy (TMVR/ViV/ViR ± PVL)	Valve implant alters PVL geometry	CT-centric planning; staged vs same-session decision; reassess PVL after valve	LVOT risk; unstable landing zone; prosthesis compromise	CT for valve planning; 3D TEE for post-implant PVL
Hostile transseptal route / alternative access	Septal closure, thick septum, extreme trajectories	Predefine access-change triggers; consider transapical (mitral) or alternative arterial access	Blind stiffness escalation; repeated prolapse; access trauma	CT/TEE to justify access and ensure coaxiality
Mixed mechanism regurgitation (PVL + transvalvular)	Plugging PVL alone may not fix hemodynamics	Mandatory mechanism confirmation; treat dominant mechanism first	Predominant transvalvular regurgitation or perforation	ASE-guided differentiation; multimodality if discordant

Table S4. Complication recognition and management during PVL closure.

Complication	Typical mechanism	Early warning signs	Prevention	Immediate bailout
Prosthetic leaflet impingement / prosthesis dysfunction	Device protrusion; malorientation; oversizing; hinge proximity	New gradient, hypotension, loss of leaflet motion (fluoro/TEE)	Multiplanar leaflet checks before release; conformable devices; avoid "oversize to seal"	Immediate recapture → reposition/resize → abort if persistent
Device embolization (acute)	Undersizing; short/irregular tract; premature release	Sudden device migration; hemodynamic change; new regurgitation	Stability ("tug") test; confirm waist purchase; favor modular strategy	Snare retrieval if safe; surgical escalation if not retrievable
Stroke / systemic embolism / air embolism	Thrombus, air, or debris mobilization	New neurologic deficit; desaturation; coronary/cerebral signs	De-airing checklist; continuous flush; maintain therapeutic ACT	Activate stroke pathway; stabilize; urgent imaging/intervention
Vascular/access complication	Large-bore sheath trauma; anticoagulation; prolonged case	Groin swelling, hypotension, falling hemoglobin	Ultrasound-guided access; pre-close when appropriate; minimize upsizing	Angio/US diagnosis; covered stent or closure; transfuse as needed
Tamponade / perforation	Wire or sheath perforation; apical injury	Hypotension; pericardial effusion on echo	Soft-to-stiff discipline; controlled sheath advancement	Pericardiocentesis; selective anticoagulation reversal; surgery if ongoing bleed
Coronary dissection/ischemia (rare)	Catheter trauma in root or ostia	ST changes; hypotension; angiographic injury	Gentle root manipulation; avoid aggressive catheter work	Coronary wiring/stenting; surgical standby
Conduction disturbance	Septal tissue interaction; device pressure	New AV block or bradycardia	Avoid bulky protrusion near septum; continuous monitoring	Temporary pacing; permanent device if persistent
Hemolysis persists/worsens	Residual high-velocity micro-jet	Rising LDH, falling hemoglobin, ongoing transfusions	Plan modular sealing; target lowest safe residual grade	Re-intervene if feasible; reassess jet/device position
